# Unplanned transfers from wards to intensive care units: how well does NEWS identify patients in need of urgent escalation of care?

**DOI:** 10.1186/s13049-025-01371-w

**Published:** 2025-06-13

**Authors:** Marianne Ask Torvik, Stig Haugset Nymo, Ståle Haugset Nymo, Eirik Hugaas Ofstad

**Affiliations:** 1 Faculty of Health Sciences, UiT Arctic University of Norway, Tromsø, Norway; 2 Division of Medicine, Nordland Hospital Trust, Bodø, Norway

**Keywords:** Early warning scores, National early warning score, Unplanned ICU transfers, Risk management, Patient safety, Hospital medicine

## Abstract

**Background:**

The National Early Warning Score (NEWS) is implemented internationally for in-hospital monitoring. It has been superior to other predictive scores, but its preventive abilities are still unclear. Additionally, data on patients who experience critical events but are not identified by NEWS as being at risk are scarce. We aimed to explore the National Early Warning Score (NEWS) as an actionable trigger to flag high-risk patients for unplanned transfers from a ward to an intensive care unit (ICU).

**Methods:**

Single-centre, retrospective study with case record reviews of all adult, unplanned ICU admissions from a ward to an ICU (level 2 and/ or level 3 ICU) for one year in a Norwegian, 200-bed, urban hospital. We examined the portion of patients flagged by a NEWS of five or seven within 24 h of an ICU transfer, if there was a change in NEWS from the previous 48 h, and how NEWS findings in this patient population differed from a general ward population.

**Results:**

Among 264 unplanned transfers from a ward to an ICU, 164 (62%) and 121 (46%) were flagged by a NEWS of five or seven, respectively. Up to 31% had a change in their NEWS, crossing the five-threshold from the previous 48 h. In contrast, nearly one in five (2077 of 11,310) of all adult admissions to the wards had at least one NEWS of five or higher, though with large variations between departments.

**Conclusion:**

NEWS did not predictably identify patients who were urgently transferred to an ICU from a ward. Less than one-third could have been identified by a recent change in their NEWS, and more than one-third did not meet the criteria of a moderately high NEWS (of five). In addition, a large portion of the ward population have NEWS of five or higher during their hospital stay. Our study emphasizes the vital role of clinical judgment in interaction with early warning scores.

**Supplementary Information:**

The online version contains supplementary material available at 10.1186/s13049-025-01371-w.

## Background

The National Early Warning Score (NEWS) was developed by the Royal College of Physicians (RCP) to improve recognition and response to clinical deterioration in hospitalized adults, aiming to prevent critical outcomes [1, 2]. Several studies have validated its ability to predict outcomes including cardiac arrest, unplanned intensive care unit (ICU) admission, and hospital death [3–7]. However, these studies have been conducted across various response systems and outcome definitions [8]. Notably, the effect of NEWS and other early warnings scores (EWSs) on preventing these outcomes is not well documented: Systematic reviews [9–13] and randomized controlled studies (RCTs) [14, 15] have not found a clear clinical benefit from NEWS or other EWSs. The systematic review from 2019 by Gerry and colleagues [10] concludes that early warning scores might even have a “*detrimental effect on patient care*,* as they might not perform as expected.”*

Nevertheless, early warning scores have an intuitive appeal: It seems only logical that prediction is preventive. Still, for a clinical decision support system like NEWS to be beneficial, it must do more than just predict or recognize deterioration. It needs to provide insights beyond what is already clinically evident and be actionable. Otherwise, it might add no value or even distract from clinical judgment. EWSs must be sensitive enough to flag most patients requiring interventions, and specific enough not to overwhelm the system with false positives, which can lead to alarm fatigue and the scores to be ignored [16–18]. Ultimately, the preventive effects of EWSs depend on the effectiveness of the institution’s response system – which again comes down to front line workers interactions with its components, for both recognition and response.

We aimed to explore NEWS as a practical decisions support system in a heterogenous hospital ward population, where NEWS is well into its post-implementation era. What is the proportion of patients in need of urgent escalation of care flagged by a high NEWS? If flagged, is this a recent change in their score, or has it been persistent over time? Moreover, how do these findings compare to findings from the general ward population? Among the outcomes NEWS is to predict, unplanned ICU admission is the least reported outcome [19], and perhaps the most site-specific. Still, considering that the prevalences of both intrahospital cardiac arrests and preventable deaths in Norwegian hospitals are low [20–22], we exclusively examined patients transferred from the wards to a higher level of care. In our data, we found that most patients who died in-hospital were either already transferred to an ICU or had limitations to escalation of care: less than 20% were still monitored with NEWS during their last 24 h, in contrast to 92% of the examined unplanned ICU transfers (including deaths at the ICUs, unpublished material). Therefore, these ward-ICU transfers should include most patients NEWS is to identify, where escalation of care is an option, and timely interventions can alter their trajectory.

Secondly, we aimed to characterize this ward-ICU patient population and their admissions, to thoroughly contextualize our findings, given the heterogeneity of both the scientific context, and hospital demographics.

## Methods

### Study design and setting

Single centre, retrospective case record reviews in a Norwegian 200-bed, urban, somatic hospital. The hospital houses 8 wards monitoring adult in-patients with NEWS: three medical, three surgical, one neurological and one short-term unit for patients independent in activities of daily living, shared by all specialities. NEWS is implemented with the RCPs guidelines for clinical response [23], giving a minimum of two complete observation sets per patient per 24 h. All wards can provide supplemental oxygen and continuous heart rhythm monitoring (cardiac telemetry), some wards (e.g., pulmonology) also offer nasal high flow oxygen, but for continuous vital sign monitoring and organ support beyond this, a higher level of care is needed, i.e., the main or intermediate ICU (IICU). The IICU corresponds to a level 2 ICU/ critical care unit [24, 25], offering continuous monitoring of vital signs including basic invasive monitoring, intensive care nursing staff with one nurse per one to two patients, vasoactive drug administration, intermittent renal replacement therapy and non-invasive ventilation. It is primarily a medical ICU, multidisciplinary attended by the internal medicine specialties, but it is also a step-up unit for all adult hospital wards, and a step-down unit from the main ICU. The main ICU serves as the regional ICU for five additional community hospitals, corresponding to a level 3 ICU / critical care unit.

### Participants and data collection

All unplanned in-hospital transfers from a somatic ward to either the ICU or the IICU among adult patients (≥ 18 years) for one year, 2019, were reviewed. Transfers were identified from a local ICU quality register, before charts were reviewed in the Norwegian electronic health record (EHR) system *DIPS* by the authors, hospital physicians in internal medicine specialties. ICU admissions directly from the emergency department (ED), following scheduled (not acute) surgery, between ICUs, or from other hospitals were excluded. Only complete NEWS observation sets were included: If a chart contained an incomplete observation set; missing at least one of the seven variables so that a score had not been calculated at the time of the observation, this observation was not included. For patients with more than one ICU transfer, the most recent was used to report patient characteristics.

Ultimately, we compared NEWS findings from the ward-ICU population to a complete ward population for one year, through an electronic NEWS database (a plug-in module to the EHR) for all adult ward admissions in our hospital for one year (February 1st, 2022, to January 31st, 2023).

### Measures

The RCP recommended trigger threshold of five for “*urgent clinical response*”, where escalation of level of care should be considered, were used to divide the ward-ICU population into two main groups: patients with no scores higher than four during the 24 prior to transfer (*NEWS24 < 5*), and patients with at least one score of five or higher (*NEWS24 ≥ 5*) Patients with a score of seven or higher, recommended by the RCP for “*emergency response”*, were also identified.

The difference between the *NEWS24*: the highest NEWS 0–24 h preceding a transfer, and the *NEWS72*: highest NEWS the previous 48 h, 25–72 h preceding a transfer, was defined as the *delta-NEWS. Delta-NEWS* was used to identify subgroups who either had stable NEWS observations over this time interval, or who crossed the trigger thresholds during the 24 h prior to transfer. The latter defined the *confirmed threshold crosser*, theoretically representing the cases where NEWS could have the most impact in guiding timely intervention. Patients who did not have a NEWS from the previous 48 h (NEWS72), but had a NEWS24 ≥ 5, were considered *possible threshold crossers*. In the general ward population, threshold crossers were defined accordingly, as patient days where a threshold crossing occurred from the previous 24 h.

Frailty and comorbidity were scored through the chart reviews, as described in detail in a previous publication [22], using the *Clinical Frailty Scale* (CFS), *Charlson’s Comorbidity Index* (CCI), and *end-stage condition* as defined by the Medicare & Medicaid Services [26].

### Statistical analysis

**Fig. 1:**
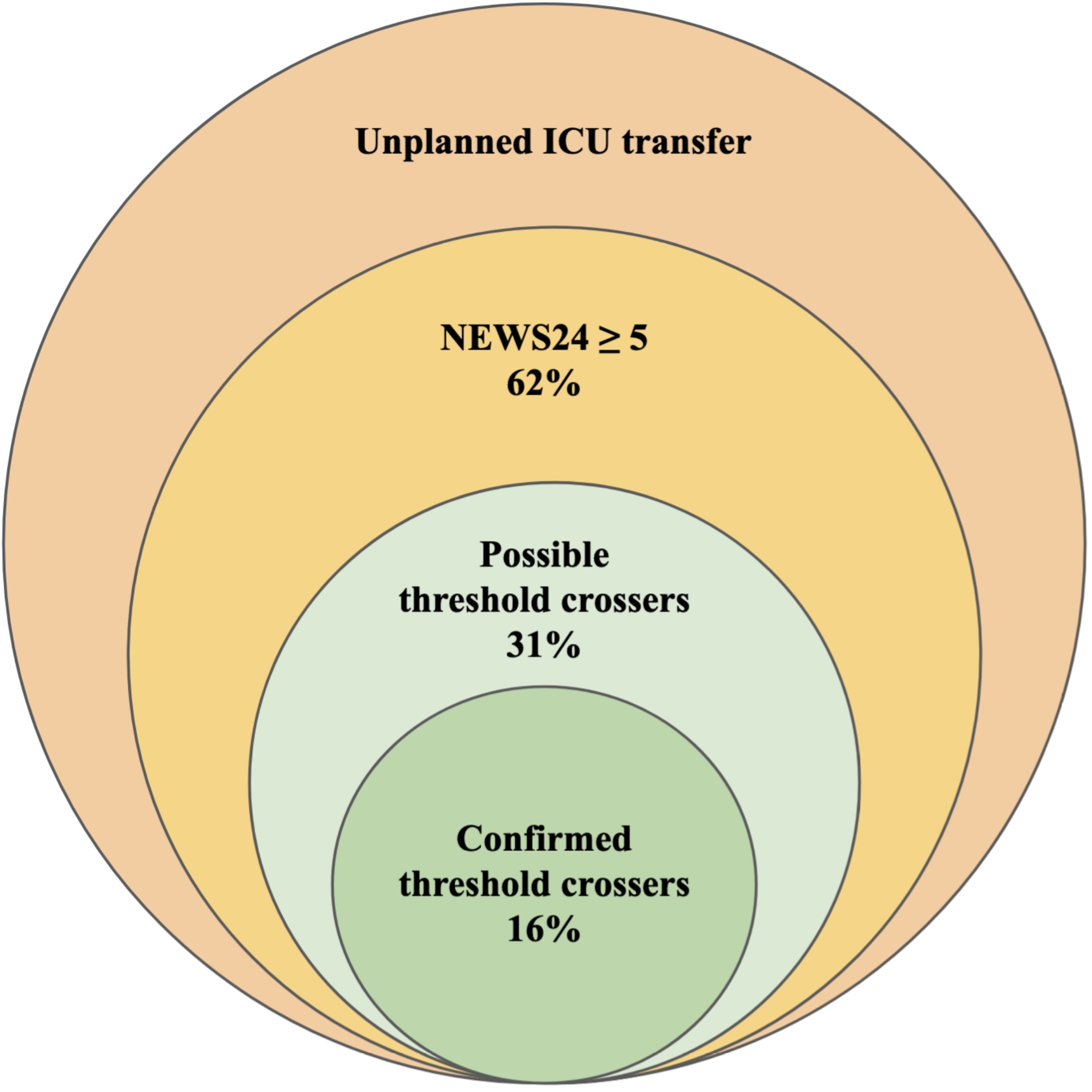
NEWS preceding an unplanned ICU transfer from a ward

Data was plotted in *Microsoft Excel* for Mac, version 18.65. Statistical analyses were performed in *Stata/MP-Parallel Edition* 18.0 for Mac. Results are reported with two sampled T-tests with unequal variances and Welch approximation. *DisplayR*, online version available October 2024, was used to draw Fig. 1.

## Results

### Participants

**Fig. 2:**
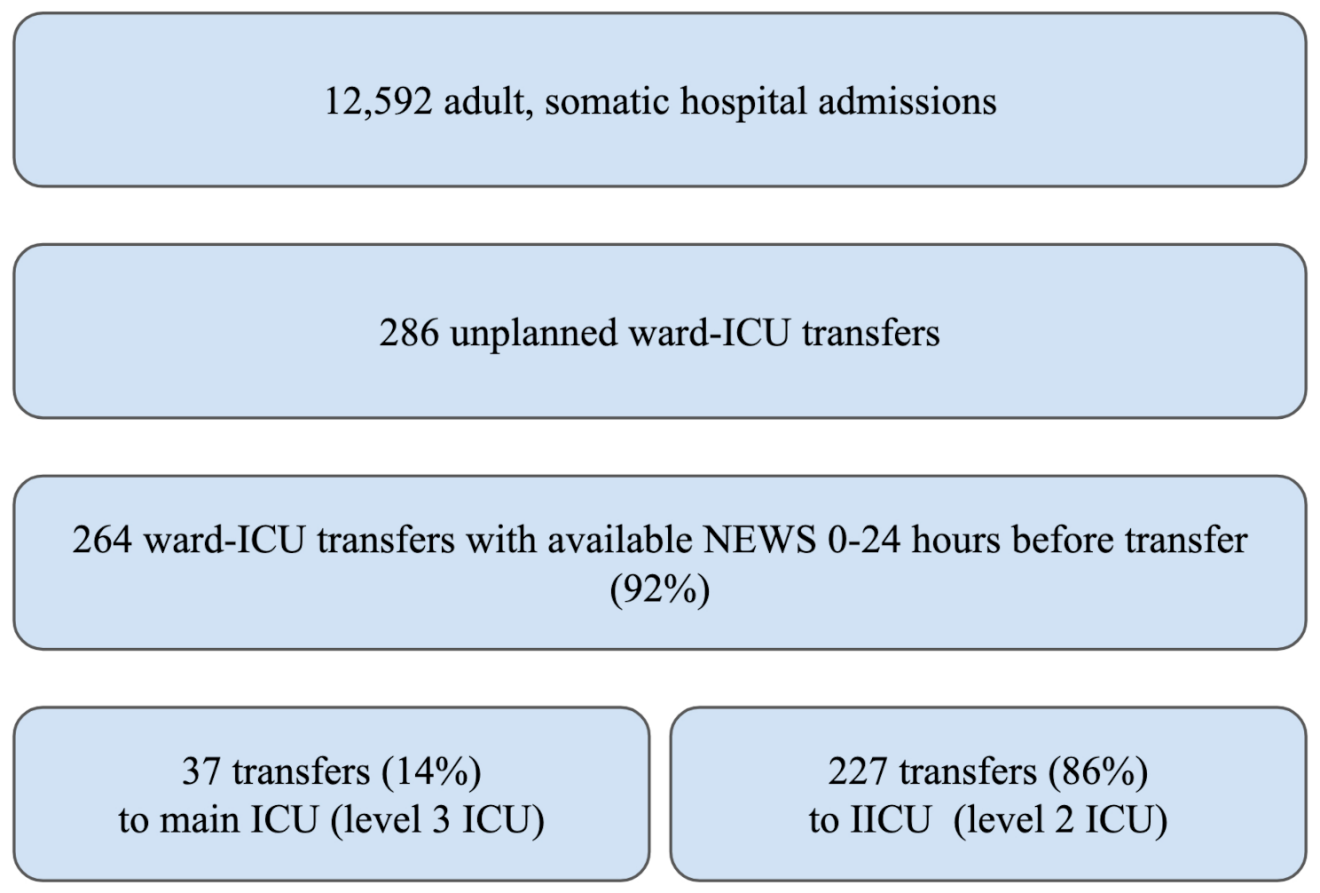
Ward-ICU population flow chart

Figure 2 presents the ICU transfer population. The event rate of unplanned ward-ICU transfer per 100 patient days was 0.54. Among 12,592 admissions, 286 unplanned ward-ICU transfers occurred in 253 admissions (2%). NEWS24 was available in 264 cases (92%), these were included for further analyses. Among the excluded 8%, transfers without at least one complete NEWS observation 0–24 h before a ward-ICU transfer, most (17 of 22) had either been in hospital for less than 24 h or were re-admissions to the ICU.

Table S1 (supplemental material) details the general hospital population and the ward-ICU population demography. If including only the main ICU, there were 37 transfers among 29 admissions (0.2%).

Unplanned ICU transfers from a ward among adults with at least one complete NEWS during the 24 h preceding the transfer were included, covering 92% of ICU transfers from the wards for one year. NEWS24: Maximum NEWS 0–24 h before ward-ICU transfer.

### NEWS preceding unplanned ward-ICU transfers

Table 1 presents NEWS preceding the ward-ICU transfers. Median NEWS24 was 6.0. NEWS24 were less than five in 38% of transfers, and less than seven in in 54%. As presented in Table 2, patients with NEWS24 of less than five had a median NEWS24 of two, if admitted to the IICU, and three if admitted to the ICU. Patients with a NEWS24 of five or higher had a median NEWS24 of 8 if admitted to the IICU, and 9 if admitted to the ICU.

NEWS72 were available in 169 transfers (69%) with available NEWS24, for whom delta-NEWS was calculated: Median delta-NEWS was 0, while 53% were zero or negative. Confirmed threshold crossers constituted 16% of the 169 transfers with available delta-NEWS. Including transfers with a NEWS24 of five or higher and no available NEWS72 as possible threshold crossers, confirmed or possible threshold crossers combined constituted 31% of all 264 ward-ICU transfers. Applying a 7-threshold for threshold crossing, 13% of all ward-ICU transfers were confirmed threshold crossers, 23% if including possible threshold crossers. This distribution is visualized in Fig. 1.

**Table 1:** NEWS preceding unplanned ward-ICU transfers

	Allward-ICU transfers	Ward- Level 3 ICU (ICU)	Ward- Level 2 ICU (IICU)
**NEWS24,*n*** (%)	***N*** = 264	***N*** = 37	***N*** = 227
Mean (median)	5.9 (6)	7.5 (8)	5.7 (6)
NEWS < 5	100 (37.9)	8 (21.6)	92 (40.5)
NEWS ≥ 5	164 (62.1)	29 (78.4)	135 (59.5)
NEWS ≥ 7	121 (45.8)	23 (62.2)	98 (43.2)
**Delta-NEWS available**,** n (%)**	*n* = 169	*n* = 25	*n* = 144
Delta NEWS mean (median)	1.3 (0)	3.6 (3)	0.9 (0)
Delta negative or zero,	89 (52.7)	5 (20.0)	84 (58.3)
Delta positive	80 (47.3)	20 (80.0)	60 (41.7)
**Threshold crossing**,** n (%)**			
***Confirmed***	*n* = 169	*n* = 25	*n* = 144
Threshold 5	27 (16.0)	10 (40.0)	17 (11.8)
Threshold 7	22 (13.0)	7 (28.0)	15 (10.4)
***Confirmed and possible***,*** combined***	*N* = 264	*N* = 37	*N* = 227
Threshold 5	82 (31.1)	19 (51.4)	63 (27.8)
Threshold 7	62 (23.5)	14 (37.8)	48 (21.1)

*NEWS24*: Maximum NEWS 0–24 h before ward-ICU transfer. *NEWS72*: Maximum NEWS 25–72 h before ward-ICU transfer. *Delta-NEWS*: NEWS24 minus NEWS72. *Confirmed threshold crosser*: NEWS72 < 5 *and* NEWS24 ≥ 5. *Possible threshold crosser*: NEWS24 ≥ 5 *and* no available NEWS72

### ICU admission and patient characteristics

Table 2 presents admission and patient characteristics. Findings are distributed between the patients flagged and not flagged by a NEWS24 of five or higher, and the threshold crossers (including both confirmed and possible). If transfers without available delta-NEWS were excluded, the distribution of patients between the three groups remained stable.

Compared to all adult hospital admissions (table S1), the ward-ICU transfer populations’ length of stay (LOS) was five times longer, and hospital mortality ten times higher. Within the ward-ICU population, several findings differed significantly, depending on their NEWS24: One was the condition leading to the ward-ICU transfer, with acute internal bleeding, arrhythmia, kidney or electrolyte disease and acute coronary syndrome (ACS) significantly more frequent among patients with a NEWS24 less than five. In contrast, sepsis and respiratory failure were significantly more frequent among patients with a NEWS24 of five or higher. Patients with NEWS24 of five or higher also had longer ICU admission and higher ICU mortality.

In general, patients admitted to both the ICU and IICU were living with considerably high age and burdens of frailty and comorbidity. Two in three patients were either 80 years or older, living with frailty corresponding to a CFS score of 6 (dependent in activities of daily living) or comorbidity with a CCI score of at least 3 (“*severe*”). This is illustrated by the one-year mortality as high as 42%. Patients with a NEWS24 of five or higher were also significantly older than those with lower scores, including a significantly higher share of patients aged 80 years or more. Comorbidity and frailty tended to be more severe among those with a NEWS24 of five or higher, but mean CCI or CFS did not differ significantly. The threshold crossers differed little from the remaining of patients with a NEWS24 of five or higher, in both admission and patient characteristics.

**Table 2:** ICU admission and patient characteristics distributed by NEWS findings

Ward-ICU transfers	All *N* = 264	NEWS24 < 5 *n* = 100	NEWS24 ≥ 5 *n* = 164	Threshold crossers *n* = 82
**Transfer condition**, ***n*** (%)				
*Respiratory failure	75 (28.4)	11 (11.0)	64 (39.0)	*25 (30.5)*
*Sepsis	41 (15.5)	7 (7.0)	34 (20.7)	*21 (25.6)*
*Arrhythmia	25 (9.5)	13 (13.0)	12 (7.3)	*6 (7.3)*
Heart failure	22 (8.3)	8 (6.0)	14 (8.5)	*7 (8.5)*
*Internal bleeding	19 (7.2)	13 (13.0)	6 (3.7)	*4 (4.9)*
Change in conscious level	18 (6.8)	8 (8.0)	10 (6.1)	*8 (9*,*8)*
*Acute coronary syndrome	15 (5.7)	13 (13.0)	2 (1.2)	*0*
*Kidney or electrolytes disease	17 (6.4)	13 (13.0)	4 (2.4)	*1 (1.2)*
Care	6 (2.3)	2 (2.0)	4 (2.4)	*3 (3.7)*
Acute abdomen	8 (3.0)	2 (2.0)	2 (1.2)	*3 (3.7)*
Cardiac arrest	3 (1.1)	1 (1.0)	2 (1.2)	*2 (2.4)*
Other	15 (5.7)	9 (9.0)	6 (3.7)	*2 (2.4)*
**NEWS24**,** mean (median)**				
IICU/ level 2 ICU	5.7 (6)	2.1 (2)	8.1 (8)	*8.6 (8)*
ICU/ level 3 ICU	7.5 (8)	2.3 (3)	8.9 (9)	*8.6 (9)*
*Transfer to,** n (%)**				
ICU, level 3 ICU	37 (14.0)	8 (8.0)	29 (17.7)	*19 (23.2)*
IICU, level 2 ICU	227 (86.0)	92 (92.0)	135 (82.3)	*63 (76.8)*
**Unique patients**	*N* = 221	*n* = 79	*n* = 142	*n* = 72
**Patient characteristics**,** n (%)**				
Male sex	130 (58.8)	52 (65.8)	78 (54.9)	*45 (62.5)*
*Age, mean (median)	71.7 (75)	67.0 (71)	74.3 (76)	*75.5 (76)*
*Age 80 or more	74 (33.5)	19 (24.1)	55 (38.7)	*31 (43.1)*
End stage condition	32 (14.5)	9 (11.4)	23 (16.2)	*8 (11.1)*
CCI, mean (median)	3.0 (2)	2.7 (2)	3.2 (2)	*3 (2)*
CFS, mean (median)	4.8 (5)	4.5 (4)	4.9 (5)	*5.0 (5)*
*Age ≥ 80, CFS ≥ 6 ***or*** CCI ≥ 3	148 (67.0)	46 (58.3)	102 (71.8)	*51 (70.8)*
**Mortality**,** n (%)**				
*ICU mortality	23 (10.4)	3 (3.8)	20 (14.1)	*9 (12.5)*
Hospital mortality	49 (22.2)	13 (16.5)	36 (25.4)	*16 (22.2)*
30-day mortality	56 (25.3)	15 (19.0)	41 (28.9)	*21 (29.2)*
One-year mortality	93 (42.1)	28 (35.4)	65 (45.8)	*33 (45.8)*

Other: Liver failure, tamponade, cytokine release syndrome, hypertension, hypotension excl. suspicion of sepsis

* Significant differences with p-values, t-test two samples with unequal variances, Welch approximation, over NEWS24 ≥ 5: Respiratory failure: 0.000. Sepsis: 0.001. ACS or arrhythmia: 0.001. Kidney failure or electrolyte disturbance: 0.004. Bleeding: 0. 012. Admission to ICU: 0. 017. Age, mean: 0.002 Age ≥ 80 years: 0.022. Age ≥ 80, CFS ≥ 6 *or* CCI ≥ 3:0.046. ICU mortality: 0.005

### ICU vs. IICU

As presented in Table 1, the vast majority (86%) of the ward-ICU transfers were to the IICU. As presented in Table 2, both mean and median NEWS24 as well as the proportions of patients with a NEWS24 of five or higher and threshold crossers were higher when a transfer was directly from a ward to the ICU, compared to the IICU. Table S2 details admission and patient characteristics between the ICU and the IICU.

### NEWS in a ward population for one year

In the electronic NEWS database, observation sets must be complete to be registered, so there was no incomplete data. 105,554 registered NEWS observation sets were registered for 11,310 admissions, with a median of two NEWS per patient per 24 h. 12,212 (12%) were five or higher in 2077 admissions (18%), 4282 (4%) were seven or higher in 998 admissions (9%). Findings varied significantly between wards: In the ward housing pulmonology, oncology and haematology, 25% of NEWS were five or higher, compared to 4% of NEWS in the ward for orthopaedic and ear-nose-throat surgery. There were 3649 confirmed or possible threshold crossers: 1773 patient confirmed threshold crossers, and 1876 possible threshold crossers.

## Discussion

We aimed to explore NEWS as a decision support tool in recognizing patients in need of urgent ICU transfers in a heterogenous hospital ward population – the landscape NEWS is implemented in internationally. We found that in over one-third (38%) of unplanned ward-ICU transfers, NEWS did not flag the patient during the last 24 h on the ward - even when using a moderate trigger threshold of five. This threshold would have flagged the remaining 62%. However, a persistently elevated NEWS is not uncommon in our clinical experience, which is why we also analysed the delta-NEWS and identified threshold crossers. Half of the ward-ICU population (53%) had a flat or negative delta-NEWS, and half of the patients identified by a NEWS of at least five (31%), were not threshold crossers. The remaining 31% of ward-ICU transfers were confirmed (16%) or possible (15%) threshold crossers. These last 31% is where NEWS most likely could aid clinicians in recognizing at-risk patients, representing a recent change in vital signs (confirmed threshold crossers), or abnormal vital signs that were not also persistent over days (possible threshold crossers). So, to summarize, in 69% of the ward-ICU transfers in our material, factors other than NEWS was likely the primary influence on the decision-making.

There are few directly comparable results to our findings, as most studies are either from emergency departments or medical assessment units (MAUs) and not general ward populations [1, 3, 4], report composite outcomes including cardiac arrests and hospital deaths combined with unplanned ICU admissions [19, 27], or include exclusively specific diagnosis populations like suspicion of sepsis [28]. Despite these differences, some parallels can be drawn. A NEWS of five or more was found in 50% of patients admitted directly from the ED to an ICU in a Dutch population [3], 66% of patients with composite outcomes in a large US ward population [27], and 75% in a large UK MAU population [1]. So, a proportion of 62% of ward-ICU transfers identified by a NEWS of five or more, appears to be to relatively consistent with other populations – albeit in a mixed context. To our knowledge, even fewer reports correspond to our delta-NEWS and threshold crosser analyses. A Finnish study [5] reported a median delta-NEWS of one for patients transferred from a ward to an ICU within the first 24 h of hospitalization, which aligns with our findings.

Further, a trigger threshold of five identified nearly one in five (18%) of all adult admissions annually in our material, but with significant variations between wards. Comparing the proportion of threshold crossers in the ward-ICU population and threshold crossing in our general ward population for one year, only about 2% of threshold crossing patient days yearly would lead to a ward-ICU transfer. These findings illustrate how the NEWS’ “one-size-fits-all” model can be troublesome: A ward where one in four NEWS is five or higher, needs different routines than one where this occurs in less than one in twenty observations. Moreover, when a NEWS of five or higher should prompt an urgent clinical evaluation, more than 12,000 evaluations would be needed to identify less than 200 transfers in a 200-bed hospital: a challenging task to identify just 62% of those requiring transfers. Increasing the threshold to seven would lead to fewer urgent calls but identify less than half (46%) of the deteriorating patients in our material. More tailored solutions between departments and patient populations still seem to be needed, as encouraged by Bedoya and colleagues [16].

In our ward-ICU population, one of the most striking features is the one-year mortality, which is as high as 42%, with little difference between admission to the IICU vs. the ICU or NEWS preceding the transfer. This high mortality is accompanied by a high burden of age, frailty, and comorbidities. Our hospital population is among those that are progressively aging and multi-morbid, where patients with one or multiple organ dysfunctions in their last years of life are a rapidly expanding patient group [29]. Such populations are potentially in high demand for intensive care - or limitations to care. Exploring and evaluating our interaction with the EWSs is no less crucial with this development in mind. Further, we found that the threshold crossers differed very little from the remaining of patients identified by NEWS of five or higher, in terms of both admission and patient characteristics. In other words, we did not identify any traits unique to this subgroup of patients where NEWS theoretically would have most impact on clinical decision making. Our findings on age and the distribution of conditions requiring ICU transfer between patients identified by NEWS or not, could illustrate that NEWS is better suited to identify respiratory failure and sepsis, which are more common in older patients, rather than other conditions, However, this could also indicate a selection bias, where younger patients are transferred more frequently and due to caution and concern, and earlier in a course of illness, compared to older patients with more comorbidities. In general, our findings could also suggest that many patients are not optimally cared for, experiencing delayed ICU transfers, potentially leading to poorer prognoses. But given relatively low rates for both ICU transfers and hospital mortality, this scenario is less likely.

Recognizing deteriorating patients is a core clinical skill of nurses and physicians that takes time and attention to develop. Studies on this clinical “intuition” or “eyeball” clinical assessment have found good predictive abilities for both mortality [30–32] and clinical deterioration on the wards [33, 34], non-inferior or superior to systematic triage or monitoring systems like EWS when compared. Adhering to mandatory systems should not hamper the development of these skills. Notably, the NEWS and other EWSs were never supposed to be a replacement but a supplement to clinical judgment [23]. However, this requires that the time and resources for both are provided. When resources are limited, time spent evaluating patients with high NEWS leaves less time to evaluate patients with lower scores, and to recognize these patients by other means.

### Limitations

This study has limitations: It is a single-centre, retrospective study with a relatively small study population. However, it does provide data beyond administrative claims methods from case records evaluated by clinicians. The general ward population database is from a different year than the ward-ICU transfer population, limiting direct comparisons and advanced statistical analyses. It still provides highly relevant contextualisation from a recent year, with stable rates of hospital and ICU admissions. As stated initially, unplanned ICU transfers should include most patients NEWS is to identify in our hospital setting. However, if NEWS *prevents* transfers to the ICUs, it benefits patients who are *not* included in our study population. As RCTs [12] have not found apparent reductions in transfers following the implementation of EWSs, the potential benefit is identifying patients earlier - rather than eliminating their need for transfers. Still, while many transfers may be inherently challenging to prevent – partly due to the demonstrated vulnerable patient population in their last years of life – another ward population is potentially identified and spared from an ICU admission by the safety net provided by NEWS. Future research could explore this possibility. Ultimately, this is mainly a study of a level 2 ICU population. Looking exclusively at patients transferred to our level 3 ICU, a NEWS of five or more identified a larger portion of ward-ICU patients (78%). Still, just half (51%) of these transfers were threshold crossers (confirmed or possible combined). Further, including both levels of ICUs, we still find ward-ICU-transfer rates that align with other comparable populations [16]. Additionally, we would argue that an ICU population including both these levels of care is of particular interest: Many patients in ours and comparable hospital populations are transferred to these, often advanced, intermediate units. Either as a final level of care due to limitations to more invasive treatment, or before a transfer to the highest level of care. Still, this group is far less studied than the general ICU population.

## Conclusions

Our findings question how actionable NEWS is for the clinicians who work with it. This highlights the importance of integrating clinical judgment with decision support tools. It is essential to enhance clinical decision support systems while simultaneously fostering the core clinical skills of front-line healthcare workers.

## Electronic supplementary material

Below is the link to the electronic supplementary material.

Supplementary Material 1

## Data Availability

Data are available on reasonable request to corresponding author.

## Abbreviations

ACS: Acute Coronary Syndrome
CCI: Charlson’s Comorbidity Index
CFS: Clinical Frailty Scale
ED: Emergency Department
EWS: Early Warning Score
ICU: Intensive Care Unit
IICU: Intermediate Intensive Care Unit
LOS: Length of Stay
MAU: Medical Assessment Unit
NEWS: National Early Warning Score
RCP: Royal College of Physicians
RCT: Randomized Controlled Trial
