# Supplementary material for: Unplanned transfers from wards to intensive care units: how well does NEWS identify patients in need of urgent escalation of care?
Source: Scand J Trauma Resusc Emerg Med. 2025 Jun 13;33:105. doi: 10.1186/s13049-025-01371-w (PMC12164093; doi:10.1186/s13049-025-01371-w)
Supplement: Supplementary file 1 — Supplementary Material 1 [file 13049_2025_1371_MOESM1_ESM.docx]

**SUPPLEMENTAL MATERIAL**

**Table S1: Adult hospital admissions and ward-ICU transfers, 2019**

| **Hospital admissions** | **N=12.592** |
| --- | --- |
| **Age group**  18-44  45-66  67-79  80-89  90+ | 16%  30%  34%  16%  4% |
| **Emergency admissions** | 64% |
| **Length of stay, mean**  All  Planned admission  Emergency admission | 4.2 days  3.6 days  4.7 days |
| **Hospital mortality**  Per admission, n=12.592  Unique patient, n= 9976 | **n=212**  1.7%  2.1% |
| **Unplanned ward-ICU transfers** | **N=286** |
| **Age group**  18-44  45-66  67-79  80-89  90+ | 5%  22%  48%  29%  5% |
| **Length of stay, mean (median)**  Hospital  ICU  Before ICU  After ICU | 20.2 (15.1)  3.0 (1.55)  6.8 (3.6)  10.3 (6.2) |
| **Hospital mortality**  ICU mortality  Hospital mortality | 10%  21% |

**Table S2: ICU vs. IICU ward-ICU transfers, 2019**

|  | **Ward-**  **Level 3 ICU**  **(ICU)** | **Ward-**  **Level 2 ICU**  **(IICU)** |
| --- | --- | --- |
| **Transfer condition, n (%)**  Respiratory failure  Sepsis  Arrhythmia  Heart failure  Bleeding  Change in conscious level  Acute coronary syndrome  Kidney or electrolyte disorder  Care  Acute abdomen  Cardiac arrest  Other* | **N=37**  9 (24.3)  7 (18.9)  0  1 (2.7)  8 (21.6)  3 (8.1)  0  1 (2.7)  0  5 (13.5)  1 (2.7)  2 (5.4) | **N=227**  66 (29.1)  34 (15.0)  25 (11.0)  21 (9.3)  11 (4.8)  15 (6.6)  15 (6.6)  16 (7.0)  6 (2.6)  3 (1.3)  2 (0.9)  13 (5.7) |
| **Mortality, n (%)**  ICU mortality  Hospital mortality  30-day mortality, any cause  1-year mortality  **Patient characteristics, n (%)**  Male sex  Age ≥ 80 years  Age, mean (median)  CCI, mean (median)  CFS, mean (median)  End stage condition | **N=29**  5 (17.2)  7 (24.1)  9 (31.0)  11 (37.9)  16 (55.2)  5 (17.2)  68.7 (73)  2.5 (2)  4.3 (4)  4 (13.8) | **N=192**  18 (9.4)  42 (21.9)  47 (24.5)  82 (42.7)  114 (59.4)  69 (35.9)  72.2 (76)  3.1 (2)  4.9 (5)  28 (14.6) |

264 ward-ICU-transfers: 37 to ICU and 227 to IICU, among 221 unique patients, 29 to ICU and 192 to IICU. Other*: Liver failure, tamponade, cytokine release syndrome, hypertension, hypotension excl. suspicion of sepsis. Abbreviations: CCI: Charlson’s Comorbidity Index. CFS: Clinical Frailty Scale.
